# Role of short-chain fatty acids in non-alcoholic fatty liver disease and potential therapeutic targets

**DOI:** 10.3389/fmicb.2025.1539972

**Published:** 2025-04-03

**Authors:** Xiang Qin, Mengyao Chen, Beihui He, Yuyan Chen, Yuelin Zheng

**Affiliations:** ^1^The First Affiliated Hospital of Zhejiang Chinese Medical University (Zhejiang Provincial Hospital of Chinese Medicine), Hangzhou, China; ^2^School of Life Sciences, Zhejiang Chinese Medical University, Hangzhou, China

**Keywords:** non-alcoholic fatty liver disease, short-chain fatty acids, gut microbiota, glycolipid metabolism, therapy

## Abstract

Non-alcoholic fatty liver disease (NAFLD) is increasing worldwide and has become the greatest potential risk for cirrhosis and hepatocellular carcinoma. The metabolites produced by the gut microbiota act as signal molecules that mediate the interaction between microorganisms and the host and have biphasic effects on human health. The gut microbiota and its metabolites, short-chain fatty acids (SCFAs), have been discovered to ameliorate many prevalent liver diseases, including NAFLD. Currently, SCFAs have attracted widespread attention as potential therapeutic targets for NAFLD, but the mechanism of action has not been fully elucidated. This article summarizes the mechanisms of short-chain fatty acids of gut microbiota metabolites to regulate the metabolism of glucose and lipid, maintain the intestinal barrier, alleviate the inflammatory response, and improve the oxidative stress to improve NAFLD, in order to provide a reference for clinical application.

## 1 Introduction

The mechanism of NAFLD as a multifactorial metabolic disease remains unclear. Currently, generally recognized is the “multiple hit” theory, which uses insulin resistance (IR) and obesity the “first hit” to increase free fatty acids (FFAs) and make the liver more susceptible to hepatotoxic injury. NAFLD progresses when multiple factors cause liver damage, including oxidative stress, mitochondrial dysfunction, inflammatory cytokines, and intestinal dysbiosis (Gou et al., 2023). The exposure of the liver to the metabolites produced by the gut microbiota through the portal vein is the direct cause of the liver metabolic disorders and degeneration and necrosis (Singh et al., 2023), which is why the gut microbiota is considered as an important entry point for the treatment of NAFLD.

Gut microbiota is a complex ecosystem composed of trillions of microbiota, which is involved in regulating body immunity, maintaining intestinal barrier integrity, and defending against foreign pathogens (Fan and Pedersen, 2021). Changing gut microbiota composition can lead to ecological imbalance and cause many diseases, including NAFLD.90% of SCFAs are produced by dietary fiber fermentation under the action of gut microbiota, and the rest are produced by dietary intake and protein metabolism. It can enter the blood through the intestine and have a direct impact on human metabolism (Hsu et al., 2024). Deng et al. (2020) study on Methionine and Choline Deficient L-Amino Acid Diet (MCD) of mice injected with SCFA solution for treatment, found that significantly reduced serum levels of alanine aminotransferase (ALT) and aspartate aminotransferase (AST) in mouse liver, Number of lipid droplets and the levels of triglycerides (TG) and total cholesterol (TC), Also reduced macrophage hepatic aggregation and proinflammatory responses induced by MCD. This indicates that the abundance of SCFAs is closely correlated with NAFLD. A growing number of studies have found that it may be because SCFAs can intervene in the process of “multiple hits.”

In this paper, we outlined the involvement of SCFAs in the metabolism of gut microbiota and explored its mechanism of action in NAFLD, hoping to find new therapeutic target for clinical treatment.

## 2 The production and metabolism of SCFAs

The precursor of most SCFAs is pyruvate, produced from glycolysis by undigested dietary fiber in the presence of the gut microbiota (Chen et al., 2020), which consists of 1–6 carbon atoms. About 50–100 mmol SCFAs are produced in the normal intestine daily, mainly acetate, propionate and butyrate, which account for 95% of all SCFAs and are present at 3:1:1 in the gut (McMurdie et al., 2022; Anachad et al., 2023).

Acetate is mainly produced by *Bifidobacteria, Lactobacilli*, and other bacteria, such as *Lachnospira, Roseburia, Akkermansia Muciniphila*, and *Parabacteroides* (Anachad et al., 2023). Besides producing most of acetate via acetyl-CoA, pyruvate synthesizes acetate via the Wood-Ljungdahl pathway. This pathway reduces CO_2_ to CO and formate, which combines with methyl groups to form acetyl-CoA (Frampton et al., 2020).

The gut microbiota can produce propionate through succinate pathway, acrylate pathway, and propanediol pathway. *Bacteroidetes* and some Negativicutes bacteria such as *Veillonella*, *Dialister*, synthesize propionate through succinate pathway (Anachad et al., 2023), and some Negativicutes bacteria via acrylate pathway, like *Megasphaera elsdenii* and *Lachnospiraceae* (Schoenfeld and Wojtczak, 2016), to generate propionate. Other bacteria, including *Escherichia coli* and *Lactobacillus reuteri* (Louis and Flint, 2017), can degrade deoxy sugars by propanediol pathway.

Butyrate is produced after condensation of acetyl-CoA through butyrate kinase pathway. In addition, acetate can be converted to butyrate via the butyryl-CoA: acetate CoA-transferase pathway (Liu et al., 2021). *Eubacterium* and *Roseburia* are the main bacteria producing butyrate, and bacteria with the transferase genes also include *Eubacterium hallii, Anaerostipes hadrus, Coprococcus catus* (Louis and Flint, 2017).

SCFAs exist mainly in the intestine in the anionic (98%) and dissociated (2%) forms (Friederike, 2018). Most of the anionic form of the SCFAs are transported through the monocarboxylate transporter 1 (MCT1) and sodium-coupled monocarboxylate transporter 1 (SMCT1), whereas the dissociated form of SCFAs are absorbed via free diffusion (Sivaprakasam et al., 2017). After absorption, SCFAs enter the liver through the portal vein to play their role. Due to efficient extraction by the liver, only a limited amount of SCFAs enter the bloodstream. The liver filters almost 100% of the butyrate and supplies 70–90% of the energy to the colonic epithelial cells, so butyrate rarely enter the circulation (Boets et al., 2017).

## 3 Changes of gut microbiota and SCFAs in NAFLD

The composition of gut microbiota in NAFLD patients is different from that of normal people. In general, patients with NAFLD have significantly decreased diversity and altered composition of gut microbiota, such as decreased *Coprococcus, Faecalibacterium, Megasphaera* and *Eubacterium*, and increased *Escherichia, Ruminococcaceae*and *Adidaminococcus,* compared to normal people (Cornejo-Pareja et al., 2024; Ha et al., 2024). Similarly, an increase in *Firmicutes* to *Bacteroidetes* (F/B) and a decrease in the abundance of *Bifidobacteria, Lactobacillus*, can also be monitored in the NAFLD rat model (Tanya et al., 2024). Among them, there are many bacteria associated with SCFA production that cause changes in SCFA levels.

Obesity is the major cause of NAFLD in children and adults, and obesity-induced ecological disturbances can lead to the development of NAFLD. The abundance of *Bacteroidetes, Gemmiger, Prevotella* and *Oscillospira* had decreased, and F/B ratio increased in obese NAFLD patients, which compared with obese youth without NAFLD (Monga Kravetz et al., 2020). In studies of obese children and adolescents, NAFLD patients have more less *Bacteroidetes* and no significant difference in *Firmicutes*. And *Faecalibacterium prausnitzii*, as the main butyrate-producing bacteria, reduced in obese NAFLD patients (Zhao et al., 2019). Of course, NAFLD not only occurs in obese people, but also high-fat diet, lack of exercise, and gender are the causes of NAFLD too (Tokuhara, 2021). In Wang’s study (Wang et al., 2016), non-obese NAFLD patients compared with healthy individuals showed a 20% increase in *Bacteroidetes* and a 24% decrease in *Firmicutes*. This is not consistent with the previous results, which suggests that the changes of gut microbiota is not single and also related to the status of NAFLD patients.

SCFAs are the key metabolites of gut microbiota to prevent NAFLD. High-fat diet-induced NAFLD mice had increased abundance of *Barnesiella, Anaerobacterium, Bacteroides, Parabacteroides*, and *Clostridium IV* after pectin supplementation in a dose-dependent manner. The levels of total SCFA, acetate and propionate elevated, with positive effects on NAFLD, which could regulate lipids, suppress oxidative stress and inflammation (Li et al., 2018). Hao et al. (2024) found that *Crataegus pinnatifida* polysaccharide (CPP) effectively reduced hepatic steatosis in NAFLD mice by decreasing the F/B ratio and increasing the abundance of *Akkermansia* to promote the production of SCFA, especially acetate and butyrate. Studies like this reveal the crucial role of the gut microbiota and SCFAs in the development of NAFLD.

## 4 Mechanisms of SCFAs to regulate NAFLD

**Figure 1:**
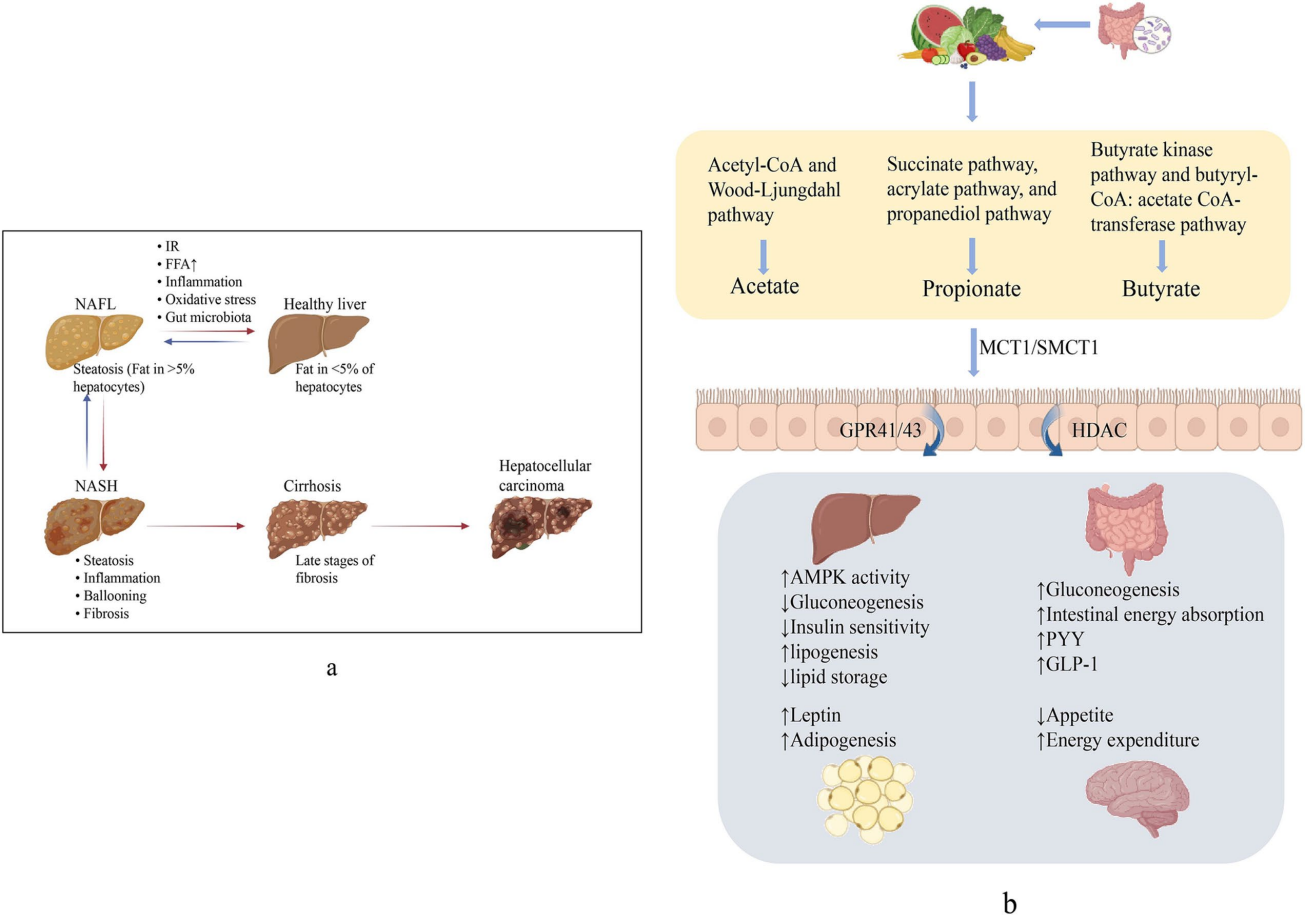
**(a)** Non-alcoholic fatty liver disease (NAFLD) spectrum. NAFL manifests as hepatic fat deposition >5% and can progress to NASH with hepatic inflammation and cellular necrosis. Broad fibrosis may lead to cirrhosis and ultimately to HCC. **(b)** Generation and role of SCFAs. SCFAs produced from dietary fiber, via active transport into the intestine mainly by SMCT1 and MCT1, activates GPCR, suppresses HDAC, and produces effects on multiple organs to perform the improvement of NAFLD.

60–70% of the energy in the gut is provided by SCFAs, and the rest flow into the liver via blood, meeting 5 to 15% of the total energy needs (Zhang et al., 2021). Only a small proportion were excreted with the feces (Canfora et al., 2015). SCFAs involved in the physiological activities of the body mainly through three G protein-coupled receptors, GPR41 (FFAR3), GPR43 (FFAR2), and GPR109A, which are variably expressed in different cells, and interfered in the occurrence and development of NAFLD by regulating glucose and lipid metabolism, restoring the intestinal barrier, and improving oxidative stress (Ikeda et al., 2022). In addition, SCFAs also inhibit the activity of histone deacetylase (HDAC) and regulates gene expression to affect body health (He et al., 2020) (Figure 1).

### 4.1 SCFAs improve the glucose and lipid metabolism disorder

Sterol-regulatory element-binding protein 1c (SREBP-1c) is a nuclear transcription factor located in the liver and can regulate the expression of related enzymes such as TG, TC and fatty acids (Lazarević et al., 2019). ACC is the rate-limiting enzyme in fatty acid synthesis. AMPK stimulated by SCFAs will inhibit SREBP-1c and ACC to reduce the synthesis of fatty acid (He et al., 2020). Activation of AMPK increases the expression of peroxisome proliferator-activated receptor *γ* coactivator 1*α* (PGC-1α) in adipose tissue and skeletal muscle, which regulates the activity of various transcription factors including peroxisome proliferator-activated receptor α (PPARα) and peroxisome proliferator-activated receptor γ (PPARγ), and promotes the oxidation of fatty acid (Wang et al., 2020). Furthermore, AMPK regulates two major enzymes in lipolysis, hormone-sensitive lipase (HSL) and adipose triglyceride lipase (ATGL) (Tang et al., 2020).

Propionate is the most important GPR43 activator, while the most potent activators of GPR41 are propionate and butyrate (Horiuchi et al., 2020). Aragonès et al. (2019) found that it could promote the secretion of Peptide YY (PYY) and glucagon-like peptide-1 (GLP-1) in intestinal L cells through the activation of GPR41 and GPR43 to affect multiple tissues, including the pancreas and the brain. PYY can inhibit intestinal peristalsis, reduce appetite and increase satiety (Psichas et al., 2015); the main function of GLP-1 is to promote the proliferation of islet *β* cell, inhibit its apoptosis, protect hepatocytes from steatosis, and regulate blood glucose by improving insulin sensitivity (Ji and Guo, 2020).

Butyrate is a potent inhibitor of HDAC and is involved in epigenetic genetic regulation. Hong’s study (Hong et al., 2016) found that short-term oral administration of butyrate could improve high fat diet (HFD) induced obesity and IR. Further exploration proved that this is presumably due to butyrate inhibiting HDAC 1 expression which activate the adiponectin-mediated downstream pathway AMPK and stimulate mitochondrial function in skeletal muscle.

### 4.2 SCFAs improve insulin sensitivity

IR is a common feature of patients with NAFLD and refers to the decreased sensitivity of the body to insulin and the reduced ability of insulin to enhance glucose intake by peripheral tissues and suppress hepatic glucose export (Song L. et al., 2022; Song Q. et al., 2022). IR causes increased peripheral lipolysis, leading to disturbed hepatic fat metabolism and inducing NAFLD. NAFLD formation can also impair the antilipolytic effects of insulin, promoting excess FFAs production, causing hepatic lipid accumulation and leading to IR (Kim et al., 2022). The vicious cycle is the basic pathological characteristics that cause liver injury and lead to lipid metabolism disorders. Mariño et al. (2017) use the non-obese diabetic (NOD) mouse model found that feeds containing acetate and propionate effectively prevented diabetes. In sterile conditions, NOD mice are more likely to develop T1D.

Phosphoenolpyruvate carboxykinase (PEPCK) and glucose-6-phosphatase (G-6-pase)are two key enzymes of gluconeogenesis, and it was found that butyrate can directly enhance the expression of these two enzymes, stimulate intestinal gluconeogenesis through cAMP dependent mechanism, increase glucose release in the intestine, reduce hepatic gluconeogenesis, and maintain glucose homeostasis (De Vadder et al., 2014). Propionate can promote gluconeogenesis through the GPR41-mediated gut-brain neural pathway (Li et al., 2017). HDAC overexpression can hinder *β* cell differentiation and inhibit the transcription of insulin gene (Yamato, 2018). Li et al. (2013) found that butyrate as a natural antagonist of HDAC could inhibit β cell apoptosis, protect βcell function and promote insulin secretion.

Apart from insulin and glucagon, leptin also plays an important role in the regulation of blood glucose. Leptin is a hormone secreted by adipose tissue that acts on receptors located in the central nervous system (Zhou et al., 2023) to regulate negative feedback on human metabolism. SCFAs promote the secretion of leptin through GPR41 and GPR43 (Li et al., 2017), acting on insulin signaling and enhancing glucose uptake in brown adipose tissue.

### 4.3 SCFAs maintain the intestinal barrier and reduce inflammation

The intestinal barrier is composed of intestinal mucosa and epithelial cells, and intercellular junctions are important parts of the intestinal epithelial barrier, including tight junctions, adhesion junctions, and desmosomes, which can prevent bacteria from entering the mucosa and destroying the immune system (Wu et al., 2022). Increased inflammatory response of intestinal mucosa and damaged intestinal epithelial cells can cause microbial translocation and induce NAFLD. Tight junction proteins are the main functional proteins of the intestinal barrier, which will enlarge the intercellular space, increase intestinal mucosal permeability to increase translocation of bacteria such as Lipopolysaccharide (LPS) to cause inflammatory response (Horowitz et al., 2023).

SCFAs activate the GPR43 pathway to stimulate potassium efflux and hyperpolarization, consequently resulting in the activation of NOD-like receptor thermal protein domain associated protein 3 (NLRP3) inflammasome, which keeps the integrity of the intestinal barrier through reparation and cell survival under stress conditions (Li et al., 2024). Butyrate appears to be the most important SCFA to regulate tight junction proteins and has been shown to improve intestinal barrier function by increasing claudin-2 through the activation of AMPK (Caetano and Castelucci, 2022). In addition, as an inhibitor of HDAC, butyrate enhanced the transcriptional activity of HIF1α, leading to higher expression of tight junction proteins to maintain intestinal barrier integrity (Fachi et al., 2019).

T regulatory cells (Tregs) can be involved in immune tolerance, suppress inflammation and allergic reactions. Gurav et al. (2015) found that the treatment of Dendritic cells (DCs) with butyrate and propionate enhanced the ability of these cells to convert T cells into Tregs. In inflamed tissues, DCs present antigen to T cells and affect T cell polarization to T helper type 1 cells (Th1), Th2 or Th17 (Nastasi et al., 2015). Akhtar et al. (2022) proposed that butyrate regulates Th17 proliferation through HDAC and induces apoptosis, thereby regulating cytokine production and maintaining intestinal symbiosis and homeostasis. Moreover, Singh et al. (2010) found that Th17, which produce IL-17, were increased in GPR109A deficient mice. This signal transduction is activated by butyrate, implying that butyrate can inhibit the production of proinflammatory factors by activating the GPCR.

The above studies have fully demonstrated the positive effect of SCFA in fighting inflammation, but butyrate also seems to have a negative effect, inducing differentiation and apoptosis in transformed cells. In the Belcheva constructed mouse model of colorectal cancer, butyrate administration induced hyperproliferation of MSH2-deficient colon epithelial cells and promoted tumor formation (Belcheva et al., 2014). This suggests that SCFA has not only a positive role in intestinal health, and its complex molecular mechanisms need further elucidation.

### 4.4 SCFAs improve oxidative stress

Oxidative stress is a state of loss of balance between the oxidative and antioxidant systems of cells and tissues, and the process leads to the overproduction of oxidative free radicals and reactive oxygen species (ROS), destroying cellular proteins, lipids and nucleic acids, leading to cellular dysfunction (Rani et al., 2016). Oxidative stress can promote mitochondrial damage, FFAs oxidation and cytokine release, a large number of hepatocyte mitochondrial dysfunction, exposing the liver to high levels of ROS which promote lipid peroxidation and hepatitis aggravation (Ziolkowska et al., 2021), directly involved in the occurrence and development of NAFLD.

Domestic and foreign studies have shown that the improvement of hepatic oxidative stress by SCFAs involves the recovery of mitochondrial function. Hu et al. (2020) demonstrated that acetate and butyrate contributed to the recovery of mitochondrial respiratory function, reduced the produced ROS, and enhanced the antioxidant capacity of the pancreatic islet cells. Moreover, acetate is more efficient in inhibiting ROS, and butyrate can reduce oxidative stress by increasing the concentration of reduced glutathione (Li et al., 2021).

## 5 Potential therapeutic targets

Up to now, clinical studies targeting SCFAs to improve NAFLD are scarce, and the direction of drug development is mostly based on the promotion of SCFA-producing advantageous microbiota, such as increasing the abundance of *Bacteroidetes, Ackermannia, Bifidobacterium, Lactobacillus*, and *Prevotella*, and decreasing the ratios of *Firmicutes* and F/B (Castillo et al., 2021). Probiotics, prebiotics and Chinese herbs are representative of commonly used clinical treatments (Chen et al., 2023; Huang et al., 2024). In addition, exercise to improve obesity is also a way to regulate NAFLD. Aerobic exercise alone can cause changes in the composition and function of human gut microbiota, increase the concentration of SCFAs in feces and the ability of gut microbiota to produce SCFAs, especially in lean people with low BMI (Allen et al., 2018). Therefore, weight loss through exercise and diet also has the effect of regulating SCFA and improving NAFLD.

Currently, a variety of probiotics, prebiotics and synbiotics containing both have been developed to improve blood lipid, reduce inflammation, enhance IR in NAFLD patients and change the composition of gut microbiota (Castillo et al., 2021). Cristofori et al. (2021) observed that commercial probiotics such as *Lactobacillus* and *Bifidobacterium*, as SCFA-producing bacteria, can promote anti-inflammation, facilitate the growth and survival of intestinal epithelial cells, and oppose pathogens by modulating the immune system and host defense. As a prebiotic, Inulin increased the abundance of *Bifidobacterium, Phascolarctobacterium* and *Blautia* while inhibited the growth of pathogenic bacteria such as *Fusobacterium* and *Corynebacterium_1* to promote the production of SCFAs, especially propionate and butyrate. The supplementation of inulin restored the integrity and function of the intestinal barrier by upregulating the expression of tight junction proteins, and effectively ameliorated the hepatic steatosis and inflammation induced by a high sucrose diet (Yang et al., 2023). Moreover, treatment of synbiotic increased the content of *Bifidobacterium* and *Faecalibacterium*, and decreased *Oscillibacter* and *Alistipes* (Scorletti et al., 2020). Ekhlasi et al. (2016) also found that synbiotic reduced serum liver enzyme levels, fasting blood glucose (FBG) and insulin levels.

Forsythoside A (FTA) is the main active ingredient isolated from Forsythiae Fructus and has prominent bioactivity. FTA can restore normal levels of *Firmicutes* and *Bacteroidetes*, elevate the abundance of *prevotellaceae_UCG-001* and *Ruminococcus_1*, and decrease the abundance of *Lactobacillus* and *mucispirillum*. With the increase of SCFA-producing bacteria, there was an increase of SCFA levels and a decrease of serum inflammatory factors lever (Fu et al., 2022).

Genistein is the main active ingredient in isoflavones and is present in almost all legumes (Jaiswal et al., 2019). Hou et al. (2023) reported that Genistein-fed mice had increased *Lactobacillus, Alloprevotella, Lachnospiraceae,* and *Bifidobacterium* content and significantly enhanced SCFA synthesis. This experiment also found that genistein inhibited the inflammatory response, reduced IL-6 and TNF-*α*, promoted the intestinal epithelial renewal in elderly animals, and effectively improved the intestinal barrier function in mice.

Oluf Pedersen (Forslund et al., 2015)’ team sequenced the gut microbiota of patients with abnormal glucose metabolism and found that metformin could significantly increase the abundance of SCFAs-producing bacteria. It have also demonstrated that under the mediation of metformin, patients have increased *Butyrivibrio, Megasphaera* and *Prevotella* levels, promoting the production of SCFAs to alter gut microbiota composition. They also found a higher abundance of *Akkermansia muciniphila, Bifidobacterium bifidum*. *Akkermansia muciniphila* as a beneficial bacterium breaks down mucins to produce SCFAs (Zheng et al., 2024) and also adheres to undifferentiated and mature enterocytes to enhance epithelial integrity (Reunanen et al., 2015). This suggests that the beneficial effects of metformin may be due to the strengthening of the intestinal mucosal barrier (de la Cuesta-Zuluaga et al., 2017).

Traditional Chinese medicine (TCM) monomer is one of the main components of TCM. As a bioactive macromolecule in many Chinese herbal medicines, polysaccharides can increase the generation of SCFAs to improve glucose and lipid metabolism by regulating gut microbiota. The Astragalus membranaceus polysaccharides (AMP) extracted from Astragalus can participate in hypoglycemic effects by improving the disturbed gut microbiota. In db/db mice treated with AMP, ratio of *Bacteroidota* to *Firmicutes* is higher, and *Allobaculum, Faecalibaculum, Akkermansia, Bifidobacterium* and *Romboutsia* positively correlated with the levels of SCFAs. It has a hypoglycemic effect and improves intestinal integrity by increasing the expression of GPR41/43 and Occludin ZO-1 (Song L. et al., 2022; Song Q. et al., 2022). Ma et al. (2022) found that *Lycium barbarum* polysaccharide (LBP) reversed the abundance of *Bacteroides, Ruminococcaceae_UCG-014, Mucispirillum* and *Intestinimonas* in HFD induced diabetic mice, and SCFAs levels were increased significantly in LBP treated mice, which corresponded to the increase in the beneficial genus, alleviating hyperglycemia and hyperlipemia. Yao et al. (2020) found that *Ruminococcus_bromii, Clostridium_methylpentosum, Roseburia_intestinalis, Clostridium_asparagiforme* and *Oscillibacter_valericigenes* increased after Cyclocarya paliurus polysaccharide (CCPP) treatment. They also found that CCPP can promote the production of SCFAs both *in vitro* and *in vivo* to control blood glucose and blood lipid. Fucoidan has been proved to increase the relative abundance of *Bacteroidetes, Ruminococcus, Prevotella,* and *Oscillospira* and to decrease the relative abundance of *Firmicutes* and *Actinobacterium*. Meanwhile, fucoidan improved blood lipids and reduced hepatic steatosis and LPS levels in dyslipidaemic rats (Chen et al., 2019).

Gegen Qinlian Decoction (GQD) is a TCM compound commonly used for the treatment of metabolic diseases, consisting of Puerariae Lobatae Radix, Coptidis Rhizoma, Scutellariae Radix, Glycyrrhizae Radix et Rhizoma Praeparata cum Melle (Lu et al., 2021). Xu et al. (2020) found that GQD can increase the expression of several bacteria, including *Faecalibacterium, Clostridium XIVa, Ruminococcus2, Butyricicoccus,* and *Coprococcus*, which are well-known butyrate producers. Therefore, they also detected increased butyrate levels in the feces. The experimental observation that GOD treatment enhances glucose clearance in rats and ameliorates systemic inflammation suggests that GOD affects host metabolism by regulating altered gut microbiota.

Intervention of diet is also one of the effective ways to improve NAFLD. High dietary fiber diets, such as those rich in fruit and legume fiber (Simpson and Campbell, 2015), are involved in the remodeling process of microbial diversity, mediating the production of the dominant microflora and delaying the progression of NAFLD development. The pectin from Citrus unshiu Marc. corrected the metabolic disturbances of SCFAs in db/db mice and reduced levels of FBG, glycated serum protein (GSP), TG, TC and low density lipoprotein cholesterol (LDL-C), while increasing levels of high density lipoprotein cholesterol (HDL-C). Ren also observed the increasing of *Firmicutes/Bacteroidetes* and the abundance of *Ligilactobacillus, Lactobacillus, Limosilactobacillus* (Yanming et al., 2023). Comparing Western-type diet (WD) and fiber-enriched Mediterranean diet (FMD), the abundance of *Firmicutes* decreased, *Bacteroidetes* and *Proteobacteria* levels increased, and SCFA content increased, especially propionate and butyrate after FMD (Soldán et al., 2024).

Fecal microbiota transplantation (FMT) can reconstruct the composition of the gut microbiota and maintain the dynamic balance of beneficial bacteria. The concentrations of the major SCFAs, including acetate, propionate, and butyrate, were increased in patients undergoing FMT (Seekatz et al., 2018). Since most patients struggle to maintain healthy lifestyle habits, NAFLD often worsen. In recent years, FMT has become a promising treatment modality for NAFLD. In the HFD-fed mouse model built by Zhou et al. (2017), the disturbed gut microbiota was corrected after FMT, with increased abundance of beneficial bacteria *Christensenellaceae* and *Lactobacillus* and decreased *Oscillibacter*. The results of this study showed that FMT can inhibit the release of IL-6 and TNF-*α*, protect the liver, and alleviate the steatohepatitis induced by HFD (Table 1).

**Table 1:** Therapeutic methods for targeting SCFAs.

Therapeutic methods	Subjects	Changes of gut microflora	Results
Probiotics	68 obese NAFLD patients	*Lactobacillus acidophilus*, *Lactobacillus rhamnosus*, *Pediococcus pentosaceus*, *Bifidobacterium lactis*, *Brevibacillus brevis* ↑	Reduce liver fat and BMI (Ahn et al., 2019)
Inulin	18 male Sprague–Dawley rats	*Bifidobacterium*, *Phascolarctobacterium*, *Blautia*↑, *Fusobacterium*, *Corynebacterium_1*↓	Restore intestinal barrier integrity, ameliorate the hepatic steatosis and inflammation (Yang et al., 2023)
Synbiotics	104 NAFLD patients	*Bifidobacterium*, *Faecalibacterium*↑, *Oscillibacter*, *Alistipes*↓	Lower body weight and liver fat (Scorletti et al., 2020)
Forsythoside A	30 C57BL/6 J mice	*Prevotellaceae_UCG-001*, *Ruminococcus_1*, *Bacteroides*↑, *Lactobacillus*, *Mucispirillum*, *Firmicutes*↓	Increase the expression of tight junction proteins and decrease the levels of LPS, MIP-1α and TNF-α (Fu et al., 2022)
Genistein	18 male C57BL/6 J mice	*Lactobacillus*, *Alloprevotella*, *Lachnospiraceae*↑	Reduce IL-6、TNF α, maintain intestinal barrier integrity, extend lifespan of mice (Hou et al., 2023)
Metformin	14 diabetic patients	*Butyrivibrio*, *Megasphaera*, *Prevotella*, *Akkermansia muciniphila*, *Bifidobacterium bifidum*↑	strengthen intestinal mucosal barrier (de la Cuesta-Zuluaga et al., 2017)
Astragalus membranaceus polysaccharides	32 db/db mice	*Bacteroidota*, *Romboutsia*, *Akkermansia*, *Faecalibaculum* ↑	Secrete GLP-1, promote the expression of Occludin and ZO-1, lower blood glucose (Song L. et al., 2022; Song Q. et al., 2022)
*Lycium barbarum* polysaccharide	60 HFD-fed diabetic mice	*Bacteroides*, *Ruminococcaceae_UCG-014*, *Mucispirillum*, *Intestinimonas*↑	Lower blood glucose and blood lipids, reduce body weight, improve glucose tolerance (Ma et al., 2022)
Cyclocarya paliurus polysaccharide	40 type 2 diabetic rats	*Ruminococcus_bromii*, *Clostridium_methylpentosum*, *Roseburia_intestinalis*, *Clostridium_asparagiforme*, *Oscillibacter_valericigenes*↑	Reduce FBG and lipids, improve HOMA-IR, OGTT and AUC (Yao et al., 2020)
Fucoidan	24 male inbred Sprague–Dawley rats	*Bacteroidetes*, *Ruminococcus*, *Prevotella*, *Oscillospira*↑, *Firmicutes*, *Actinobacterium*↓	Improve blood lipids, reduce hepatic steatosis and LPS levels (Chen et al., 2019)
Gegen Qinlian Decoction	30 type 2 diabetic rats	*Faecalibacterium*, *Clostridium XIVa*, *Ruminococcus2*, *Butyricicoccus*, *Coprococcus*↑	Improve glucose clearance and HOMA-IR, prevent obesity (Xu et al., 2020)
Pectin from Citrus unshiu Marc.	18 db/db mice	*Ligilactobacillus*, *Lactobacillus*, *Limosilactobacillus*↑	Lower FBG, GSP, TC, TG, and LDL-C, increase HDL-C (Yanming et al., 2023)
Fiber-enriched Mediterranean diet	20 healthy volunteers	*A. Butyriciproducen*, *A. hadrus*↑	Increase gut microbial metabolic β diversity (Barber et al., 2021)
Fecal microbiota transplantation	36 HFD-fed mice	*Christensenellaceae*, *Lactobacillus*↑, *Oscillibacter*↓	Improve steatosis, decrease TNF- α, MCP-1, IL-1, and IL-6 (Zhou et al., 2017)

## 6 Summary

Currently, the role of gut microbiota in the development of NAFLD has become a research hotspot, and the regulation of gut microbiota is a novel target for the treatment of NAFLD. The gut microbiota metabolite SCFAs are one of the effective targets for ameliorating NAFLD. Although SCFA are seldom used directly in clinical trials, there have numerous researches showing the correlation between SCFAs and NAFLD in recent years. This review summarizes the therapeutic role of SCFAs for NAFLD by regulating glucose and lipid metabolism, improving IR, anti-inflammation, anti-oxidative stress, and improving intestinal barrier function. Increasing the content of beneficial flora and metabolite SCFAs through special diet or probiotics to restore liver metabolism is an effective method and a new idea for the treatment of NAFLD. However, the role of SCFAs is still controversial. Its effect mechanism is complex, and it will harm the human body when the concentration is too high. But in general, the impact on the host is more beneficial than harmful. Therefore, the research on the relationship between SCFAs and the human body still needs to be continued to achieve precise treatment.
